# A *Nuphar lutea* plant active ingredient, 6,6′-dihydroxythiobinupharidine, ameliorates kidney damage and inflammation in a mouse model of chronic kidney disease

**DOI:** 10.1038/s41598-024-58055-1

**Published:** 2024-03-30

**Authors:** Daniel Landau, Jannat Khalilia, Eden Arazi, Ana Foigelman Tobar, Daniel Benharroch, Avi Golan-Goldhirsh, Jacob Gopas, Yael Segev

**Affiliations:** 1 Department of Nephrology, Schneider Children’s Medical Center, Petah Tikva, Israel; 2 School of Medicine, Tel Aviv University, Tel Aviv, Israel; 3 Shraga Segal Department of Microbiology Immunology and Genetics, Faculty of Health Sciences, Ben Gurion University of the Negev, Beer Sheva, Israel; 4 Pathology Department, Rabin Medical Center, Petach Tikva, Israel; 5 Department of Pathology, Soroka University Medical Center and Faculty of Health Sciences, Ben-Gurion University of the Negev, Beer Sheva, Israel; 6 The Jacob Blaustein Institutes for Desert Research (BIDR), French Associates Institute for Agriculture and Biotechnology of Drylands, Ben-Gurion University of the Negev, Sede Boqer Campus, Beer Sheva, Israel

**Keywords:** Kidney diseases, Chronic inflammation, Diseases

## Abstract

Chronic Kidney Disease (CKD) associated complications are associated with increased inflammation through the innate immune response, which can be modulated with anti-inflammatory agents. An active ingredient derived from the *Nuphar lutea* aquatic plant, 6,6′-dihydroxythiobinupharidine (DTBN) has anti-inflammatory properties, mainly through the inhibition of NF-κB. We tested the effects of DTBN on mice with CKD. After preliminary safety and dosing experiments, we exposed 8 weeks old male C57BL/6J mice to adenine diet to induce CKD. Control and CKD animals were treated with IP injections of DTBN (25 μg QOD) or saline and sacrificed after 8 weeks. Serum urea and creatinine were significantly decreased in CKD-DTBN Vs CKD mice. Kidney histology showed a decrease in F4/80 positive macrophage infiltration, damaged renal area, as well as decreased kidney TGF-β in CKD-DTBN Vs CKD mice. Kidney inflammation indices (IL-1β, IL-6 and P-STAT3) were significantly decreased in CKD-DTBN as compared to CKD mice. DTBN treatment showed no apparent damage to tissues in control mice, besides a decrease in weight gain and mild hypoalbuminemia without proteinuria. Thus, DTBN significantly improved renal failure and inflammation indices in CKD mice. Therefore, this and similar substances may be considered as an additional treatment in CKD patients.

## Introduction

Chronic kidney disease (CKD), like other chronic non-infectious conditions, is a state of low-grade inflammation through activation of the innate immune system^1^. Clinical and experimental data show that increased inflammation is associated with CKD progression^2^ and complications, including erythropoietin resistant anemia^3^ and cardiovascular events^4^ CKD associated increased inflammation can be modulated with anti-inflammatory agents. We have recently described the beneficial effects of IL1 inhibition in a mouse model of CKD for both anemia and CKD severity^5^. More recently, we have shown the beneficial effects of colchicine on kidney damage parameters, as well as kidney inflammation and fibrosis, in a short term (3 weeks) kidney damage model^6^.

**Figure 1:**
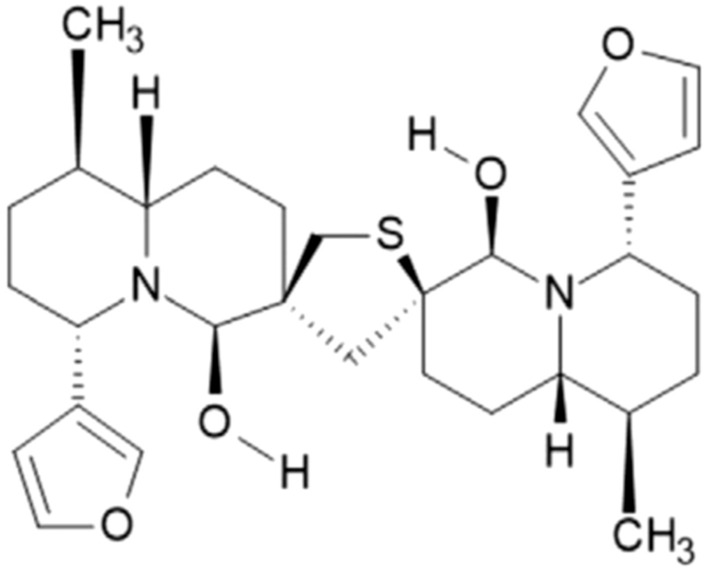
Structure of 6,6′-dihydroxythiobinupharidine (DTBN).

We have previously published the ability of an alkaloid semi-purified mixture extracted from the *Nuphar lutea* which consists mainly of dimeric sesquiterpene thioalkaloids called thiobinupharidines and thiobinuphlutidines, to have potential therapeutic use. The extract from *Nuphar lutea* as well as a purified molecule from this extract, 6,6′-dihydroxythiobinupharidine (DTBN) (Fig. 1), are pleiotropic in their action. The semi-purified extract has anti-inflammatory activity by downregulating NF-κB^7,8^ and partially preventing LPS-induced septic shock and peritonitis^7^. It is also effective against free as well as intracellular *Leishmania major* parasites^9–11^. The extract has anti-metastatic properties synergistically with conventional chemotherapy drugs, both in vitro and in vivo^12^.

Purified DTBN primes neutrophils, enhances phagocytosis, reactive oxygen species (ROS) production, and NET formation^13^. We also reported in-vitro antileukemic effects of DTBN by induction of apoptosis, correlated with significant biphasic changes in ROS cytosolic levels^14^. We have recently published that DTBN has in vitro and in vivo therapeutic potential against SARS-CoV-2^15^. Regarding molecular targets, DTBN very efficiently and covalently, inhibits human type II topoisomerase^16^, conventional protein kinase C (PKC) molecules, most efficiently PKC alpha and PKC gamma^17^, as well as inhibiting cysteine proteases such as cathepsins S,B and L and papain^18^.

Since CKD associated complications, such as progression and anemia are associated with increased inflammation through the innate immune response, we tested the anti-inflammatory effects of DTBN, asking whether DTBN will significantly improve renal failure indices and inflammation in CKD mice.

## Results

Experimental groups included wild type mice on a control regular (C) or adenine diet (CKD), DTBN treated mice on a regular (C-DTBN) or adenine diet (CKD-DTBN). There was no increased mortality among the DTBN treated animals. DTBN treatment showed no apparent biochemical (supplementary Fig. S3) or histological damage to tissues in control mice (supplementary Fig. S4). However, a decrease in weight gain and mild hypoalbuminemia (without proteinuria) were seen in C-DTBN Vs C (supplementary Figs. S1 and S2).

**Figure 2:**
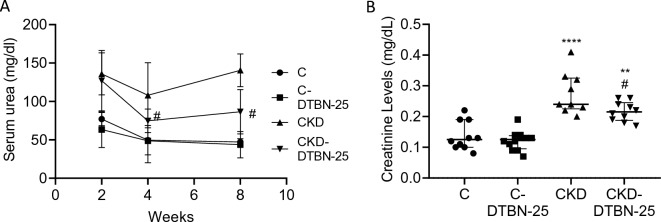
DTBN treatment improved serum urea (**A**) and creatinine (**B**) in CKD-DTBN mice. Experimental groups include wild type mice on a regular (C) or adenine diet (CKD), DTBN treated mice on regular diet (C-DTBN) or adenine (CKD-DTBN) (n = 6–12). #: p < 0.05 Vs CKD; **: p < 0.005 Vs controls; ****: p < 0.0001 Vs controls. Urea levels were obtained along the experiment and creatinine- at sacrifice.

Serum urea was followed during the experiment and increased over time in the adenine treated CKD mice. DTBN treatment improved serum urea in CKD-DTBN treated mice. Serum urea was reduced by 1.3 fold after 4 weeks of treatment (p < 0.05) and by more than 1.4 fold after 8 weeks of treatment (p < 0.05, Fig. 2A). Serum creatinine (Fig. 2B) was also reduced by almost 1.3 fold in CKD DTBN treated mice as compared to untreated CKD mice. Blood counts revealed mild anemia, and increased leucocyte counts in both CKD groups Vs controls, without difference between them (Supplementary Fig. S7).

**Figure 3:**
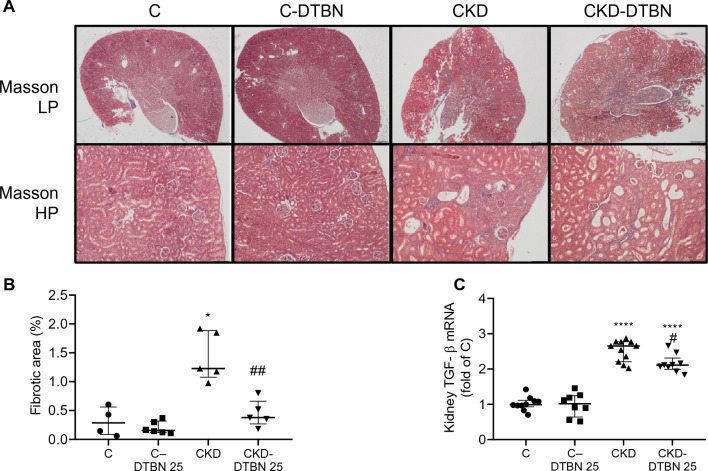
Histological analysis. Experimental groups include wild type mice on a regular (C) or adenine diet (CKD), DTBN treated mice on regular diet (C-DTBN) or adenine (CKD-DTBN). (**A**) Renal phenotype: representative kidney sections: panoramic low power (LP) view of kidneys (X40) shows depression of the cortical contours in the CKD groups that correspond to areas of tubular atrophy. Higher magnification (HP) show mild decrease in inflammatory response in the tubulointerstitium in CKD-DTBN Vs CKD. (**B**) Kidney atrophic area percentage as determined by image analysis (n = 4–5); and (**C**) TGF β mRNA (n = 8–11). *: p < 0.05 Vs controls; ****: p < 0.0001 Vs controls; ##: p < 0.005 Vs CKD.

**Figure 4:**
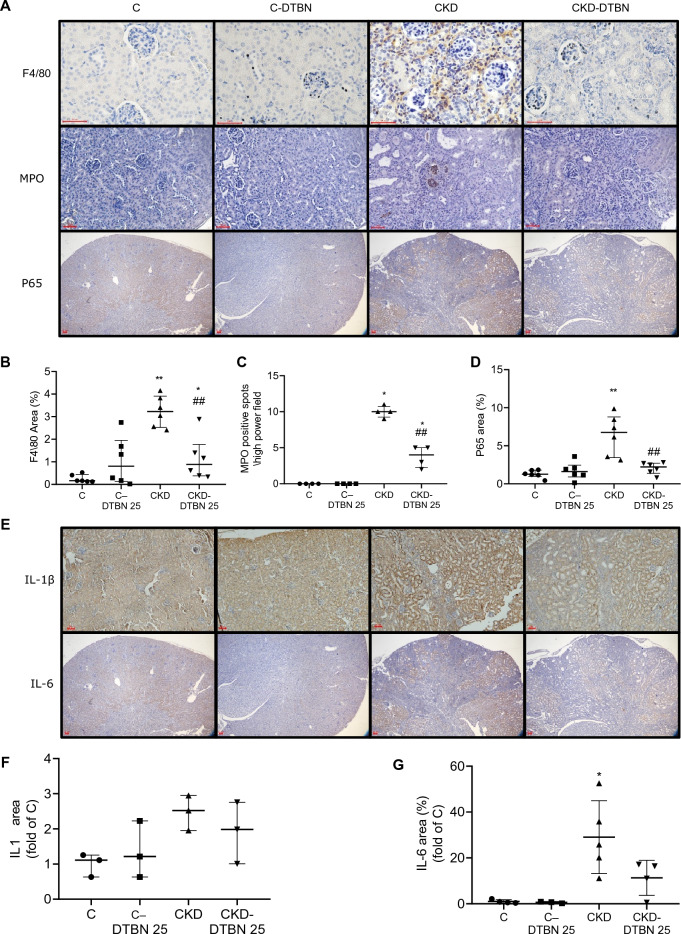
(**A**) Immunohistochemical (IHC) staining for macrophages (F4/80) (n = 6), MPO (a neutrophil marker) (n = 4), p65 (a marker of NF-kB activation) (n = 6), (**B**–**D**): IHC antibody staining extent of F4/80, MPO and P65. (**E**) IHC for IL-1 β and IL-6 and its quantitation (F-G), analyzed with the *ImageJ* software. Two to ten X40 magnification fields were quantified for each animal (3–5 animals/group). Red bar = 50 µm. *: p < 0.05, Vs controls; ** p < 0.005 Vs controls; ****: p < 0.0001 Vs controls. ## p < 0.005 Vs CKD. ###: p < 0.001 Vs CKD.

Adenine diet caused crystal deposition (which are washed by tissue fixation) and tubulointerstitial inflammation. Cortical wedge-like areas with beginning of tubular atrophy were seen, leading to an irregular contour of the cortex edge. Dilated tubules with polymorphonuclear cells (MPO positive) casts were seen in all renal parenchyma. Interstitial inflammation was accompanied by an increase in tubular atrophy and mild fibrosis. In the CKD-DTBN animals no marked differences in the extent of tubular atrophy or wedge-like areas could be detected, but the number of tubules with casts of polymorphonuclear cells was reduced in this group (Figs. 3A, 4A). The atrophic areas in the CKD group were more than 4.5-fold higher than controls. DTBN reduced this atrophic area extent by three-fold as compared to CKD mice (from 1.4 to 0.4%, p < 0.01) (Fig. 3B). In addition, kidney TGFβ mRNA, a marker of fibrosis, was decreased in CKD-DTBN Vs CKD (Fig. 3C). Macrophage infiltration, as measured by F4/80 area was reduced by 2.8-fold (1.1% in CKD-DTBN Vs 3.2% in CKD, p < 0.01) (Fig. 4B). Neutrophils cluster spot numbers were reduced by almost three-fold in the CKD-DTBN group as compared to CKD mice (3.75 Vs 10 times of control respectively, p < 0.05) (Fig. 4C).

NF-κB is a central transcription factor activated in inflammation. We stained and quantitated its p65 subunit. DTBN treatment to CKD animals reduced its presence by three-fold (2.1% and 6.4% in CKD-DTBN Vs CKD respectively, p < 0.01) (Fig. 4D). IL-1β and IL-6 staining (Fig. 4E) was reduced in DTBN treated CKD mice by 1.3 and 2.6 fold respectively comparing to CKD untreated mice (Fig. 4F–G).

**Figure 5:**
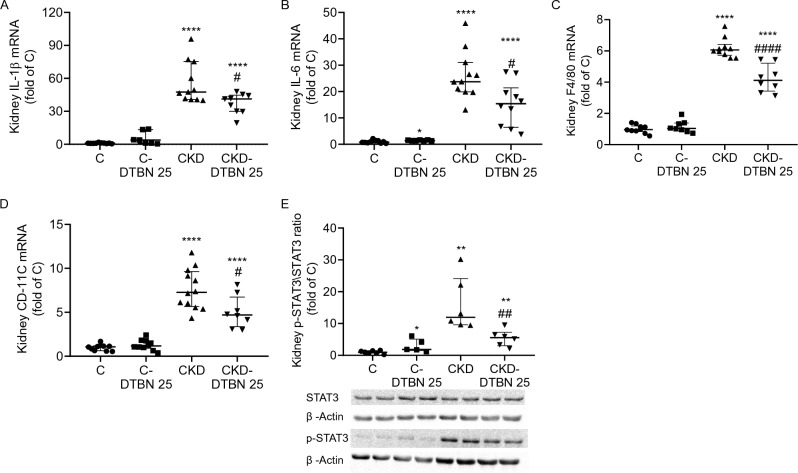
Kidney inflammation markers: (**A**) IL-1β mRNA (n = 6–10); (**B**) IL-6 mRNA (n = 5–9); (**C**) F4/80 mRNA (n = 8–10); (**D**) CD11c mRNA (n = 8–12); (**E**) pSTAT3/STAT3 protein ratio (n = 5–7). Experimental groups include wild type mice on a regular (**C**) or adenine diet (CKD), DTBN treated mice on regular diet (C-DTBN) or adenine (CKD-DTBN). ** p < 0.005 Vs controls; ****: p < 0.0001 Vs controls. #: p < 0.05 Vs CKD; ## p < 0.005 Vs CKD. ####: p < 0.0001 Vs CKD.

In addition to the NF-κB, IL-1β and IL-6 staining, we determined pro-inflammatory markers by qRT-PCR. IL-1β mRNA levels (Fig. 5A) in CKD-DTNB treated mice was reduced by 1.4-fold as compared to CKD mice (37.3 and 51.9 fold of C respectively, p < 0.05). IL-6 mRNA levels (Fig. 5B) were reduced by 1.6 fold in CKD-DTBN mice as compared to CKD mice (25.2 and 15.7 fold of control respectively, p < 0.05). Consistently with the immunohistochemical staining in Fig. 4, F4/80 mRNA levels (Fig. 5C) were also reduced in CKD-DTBN treated mice (4.23 vs. 6.19-fold of control p < 0.001).

CD11c is expressed in several cell types and serves as a marker of dendritic cells, which have been found to respond in murine models of chronic nephritis^24^. CD11c mRNA levels were reduced by 1.5 fold in CKD-DTBN treated mice as compared to CKD mice (5-fold and 7.6 fold of C respectively, p < 0.05) (Fig. 5D).

The signal transduction of the IL-6 family of cytokines is dominated by signal transducer and activator of transcription 3 (STAT3), which has been proposed as a target molecule to reduce fibrosis^25,26^. In our study p-STAT3/STAT3 protein ratio was reduced by 2.9 fold in CKD-DTBN as compared to CKD mice (5.4 vs. 15.9 fold of C, p < 0.01) (Fig. 5E).

## Discussion

CKD is a condition of persistent low-grade inflammation, through activation of the innate immune system. This may be associated with adverse long-term complications, such as increased risk for cardiovascular disease, anemia and progression of CKD itself. In this study (still designed as a prevention and not as an intervention study) we show beneficial effects of DTBN on kidney inflammation, as well as kidney damage and fibrosis, using a mouse model of tubulointerstitial kidney disease by adenine diet. Contrary to our previous study, where adenine exposure was for only 3 weeks and colchicine was the tested anti-inflammatory agent, we show in the current longer term study beneficial effects of DTBN on serum urea and creatinine levels, as well as kidney fibrosis and macrophage infiltration.

DTBN or the whole *Nuphar* extract have been tested previously in several animal models, including a model of LPS induced cytokine storm^8^, melanoma antimetastatic effect^12^ and a model of SARS-Cov-2 induced pneumonitis^15^. However, it has not been tested yet in humans. Therefore, potential safety issues may raise. We have performed several preliminary dosing tests and have not found any evidence of toxic damage (by survival and histology) to the major organs (brain, heart, liver, intestines, spleen). Liver CRP and serum SGOT were not elevated in C-DTBN Vs C mice. Therefore, the decrease in serum albumin in association with decreased weight gain in the C-DTBN group, together with the lack of proteinuria may still suggest a minor liver damage not yet observed by histology^19^.

In our study we show decreased inflammation as well as decreased renal fibrosis in DTBN treated CKD mice. The inflammatory pattern was different for polymorphonuclears (MPO positive cells), which localized to tubular lumen, in contrast to macrophages (F4/80 positive cells) which were more spread along the tubulointerstitium. The anti-inflammatory pattern induced by DTBN included a decrease in IL1 and IL6 as well as p65. We have previously reported on beneficial effects of colchicine on kidney injury parameters, including polymorphonuclear cell accumulation and macrophage infiltration, in a short term (3 weeks) adenine diet intervention^6^, in association with tubulin polymerization inhibition, which could affect polymorphonuclear migration. Interestingly, a DTBN variant has been also shown to have properties of inhibition of tubulin polymerization: it decreased expression of LIMK1 and thus the levels of phosphorylated Cofilin. As a result, actin regulation is disrupted, and cell migration is inhibited^20^.

The histological consequence of DTBN's effects in CKD can be seen for its ability to prevent macrophage recruitment. A similar anti-inflammatory effect by DTBN or the nuphar extract was previously reported by us, including NF-κB inhibition^7^ and reduction of proinflammatory cytokine response in macrophages and in sera of LPS treated mice^8^. In addition, the nuphar extract has been shown to inhibit NF-κB in leishmania infected macrophages^11^. In this study, the extent of kidney damage in the non-treated CKD group, which was more than 4.5-fold higher than controls, was significantly reduced with DTBN treatment, in association with a decrease in key proinflammatory mediators (IL1, IL6, NFkB and STAT3).

CD11c is expressed in several cells and serves as a marker to identify dendritic cells. Kidney dendritic cells form an abundant network in the renal tubulointerstitium and constantly survey the environment for signs of injury or infection to alert the immune system to the need to initiate defensive action. Recent studies have identified a role for dendritic cells in several murine models of acute renal injury and chronic nephritis^21^. CD11c mRNA levels were also reduced in CKD-DTBN treated mice as compared to CKD mice.

The signal transduction of the IL-6 family of cytokines is dominated by signal transducer and activator of transcription 3 (STAT3). In addition, STAT3 has been proposed as a target molecule to reduce fibrosis^22,23^. Previous studies have shown the beneficiary effects of non-steroidal anti-inflammatory agents, such as colchicine^24^ and canakinumab^25^, in the prevention of cardiovascular complications in humans. Colchicine was also shown to inhibit kidney injury in a short-term adenine intervention study^6^. In addition, thalidomide has shown beneficiary effects in an animal model of CKD^26^. Similar findings have been reported using sodium copper chlorophyllin, an USFDA approved dye with anti-oxidant activities^27^. Recently, an anti-IL-6 monoclonal antibody was shown to decrease inflammation markers and improve anemia in a subgroup of hemodialysis patients with elevated circulating IL6 due to a genetic tendency^28^. This finding has now been approved in a follow up study of patients with CKD 3-5 and elevated CRP^29^. It remains to be determined whether these beneficiary effects by anti inflammatory agents on anemia and cardiovascular complications will also be shown for CKD progression in humans. For example, in patients with rheumatoid arthritis, increased inflammation through the IL1 pathway is associated with CKD progression^30^.

In conclusion, DTBN significantly improved renal failure indices and inflammation in CKD mice. Therefore, this or similar substances may be developed as an additional treatment in CKD patients.

## Methods

### Animals

This study was approved by the Ben-Gurion University of the Negev Animal Use and Care Committee, protocol number IL-39-07-2018(D). All protocols comply with the NIH Guidelines and were reported in accordance with ARRIVE guidelines (https://arriveguidelines.org). Animals were housed in standard laboratory cages. Food and water were given ad libitum. After preliminary safety and dosing experiments: we used 8 weeks old male C57BL/6J mice, and provided them with an Adenine diet (0.3% for 9 days, then 0.2% for the rest of time) to induce CKD^31^. Mice (Harlan Laboratories Inc. Rehovot, Israel) were treated with IP injections of a non-toxic dose of DTBN (25 ug QOD) or saline, forming 4 experimental groups: control (C), CKD, C-DTBN and CKD-DTBN. CKD was induced by adenine diet for 8 weeks as follows: first, a 0.3% adenine, 0.9% phosphorus and 75 ppm iron diet was given for 9 days, and then switched to a 0.2% adenine diet and unchanged phosphorus and iron until the end of the experiment. Control groups were fed with a control diet (0.3% phosphorus, ~ 75 ppm iron). All diets were purchased from Envigo Teklad, (Huntingdon, UK).

6,6′-Dihydroxythiobinupharidine (DTBN, 30343-70-5) (Fig. 1) was purchased from Sigma/Merck (SMB00609), was diluted in DMSO to a 1 mg/ml stock solution and further diluted in saline. Intra peritoneal (i.p.) injections of a working solution containing 25 µl DTBN stock, 7.5 µl DMSO and 67.5 µl saline (25 μg/0.1 ml) were given three times a week for 8 weeks. Mice were sacrificed at experiment end, using lethal anesthesia with ketamine and xylazine, collecting: blood, and liver and kidney tissues.

### Blood analyses

Tail blood samples for complete blood count (CBC) and urea were taken two times during experiment and prior to sacrifice. Blood counts were determined using a veterinary heamatology analyzer (Exigo H400, Boule, FL, USA). Tail blood urea was analyzed using an enzyme linked immunosorbent (ELISA) commercial kit (Abcam, Cambridge UK). Serum creatinine, was analyzed using the AU2700 analyzer (Beckman-Coulter, CA, USA), based on the Jaffe method for determination of mouse serum creatinine. Urine albumin was tested by the Coomassie blue reaction on urine samples that were loaded on an SDS-PAGE. Bovine serum albumin (fraction V, MP biomedicals, OH, USA) served as control.

### RNA extraction and real time PCR

RNA was extracted using a commercial kit (Macherey–Nagel, Dueren, Germany). cDNA was transcribed using qScript cDNA synthesis kit (Quanta biosciences, MD, USA) and qPCR assays were performed with power SYBR green PCR master mix (Applied Biosystems, Foster City, CA, USA) as previously described^32^, using the ABI Prism 7300 sequence detection system (Applied Biosystems, CA, USA). Primers for quantification (Sigma-Aldrich, Rehovot, Israel) are summarized in Table 1.

**Table 1:** Primers used for RT-PCR.

Gene	Sense-forward	Anti-sense-reverse
TGF-β	GCAACATGTGGAACTCTACCAGA	GACGTCAAAAGACAGCCACTCA
IL-1β	ACAACCACGGCCTTCCCTACTT	CACGATTTCCCAGAGAACATGTG
IL-6	CTATACCACTTCACAAGTCGGAGG	TGCACAACTCTTTTCTCATTTCC
F4/80	CGTCAGGTACGGGATGAATATAAG	CTATGCCATCCACTTCCAAGAT
CD-11c	CTGGATAGCCTTTCTTCTGCTG	GCACACTGTGTCCGAACTC
β-actin	GGTCTCAAACATGATCTGGG	GGGTCAGAAGAATTCCTATG
CRP	CCATTTCTACACTGCTCTGAGCAC	CCAAAATATGAGAATGTCGTTAGAGTTC

### Western Immunoblot analysis

The following antibodies were used for evaluation of the kidney extracts: β-actin (MP Biomedicals, OH, USA), STAT3 and pSTAT3 (Cell Signaling Technology, MA, USA), as previously described^33^.

### Kidney histology

Kidney segments were fixed in 4% formalin for 48 h, then embedded in paraffin and cut. Kidney sections were deparaffinized, rehydrated and stained with Masson’s trichrome (Bio-Optica, Milano, Italy). Histological analyses were done by two Pathologists (AFT and DB) blinded to study groups. Damaged area quantification of Masson’s trichrome staining was performed as described by Chen et al. using the *ImageJ* software^34^.

Immunohistochemistry staining was performed as previously described^5^. Primary antibodies against F4/80 (BM-8) (diluted 1:30; Santa Cruz biotechnology Inc., Dallas Texas, USA) and Myeloperoxidase (MPO) (diluted 1:50; Abcam, Cambridge, UK), NF-κB p65 (Bio-Rad, CA, USA), IL-1 and IL-6 (GeneTex, CA, USA) were used for the immunohistochemistry staining. For image processing, Topika Analysis software (Topika, Tel Aviv, Israel) was used. Area quantification of immunohistochemical staining was performed using an *ImageJ* software as described.

### Data analysis

Since our data do not comply with parametric test like ANOVA, we used the comparable non-parametric test Kruskal–Wallis to compare multiple groups, and the non-parametric test Mann–Whitney for comparison of each two groups. The null hypotheses were rejected at the 5% level. Values along the manuscript are presented as medians ± interquartile range (IQR). Asterisks indicate a significant difference between the indicated and the control group, where hash marks indicate a significant difference between the indicated and the CKD group. ****/#### indicates p value < 0.0001, ***/### indicates p value < 0.001, **/## indicates p value < 0.005, */# indicates p value < 0.05.

### Supplementary Information

Supplementary Figure S1.

Supplementary Figure S2.

Supplementary Figure S3.

Supplementary Figure S4.

Supplementary Figure S5.

Supplementary Figure S6.

Supplementary Figure S7.

## Data Availability

The study protocol has been submitted to the *Preclinicaltrials.eu* website (protocol # PCTE0000405). The data underlying this article will be shared on reasonable request to the corresponding author.
