# Supplementary figures and images for: A Nuphar lutea plant active ingredient, 6,6′-dihydroxythiobinupharidine, ameliorates kidney damage and inflammation in a mouse model of chronic kidney disease
Source: Sci Rep. 2024 Mar 30;14:7577. doi: 10.1038/s41598-024-58055-1 (PMC10981724; doi:10.1038/s41598-024-58055-1)

## Slide 1
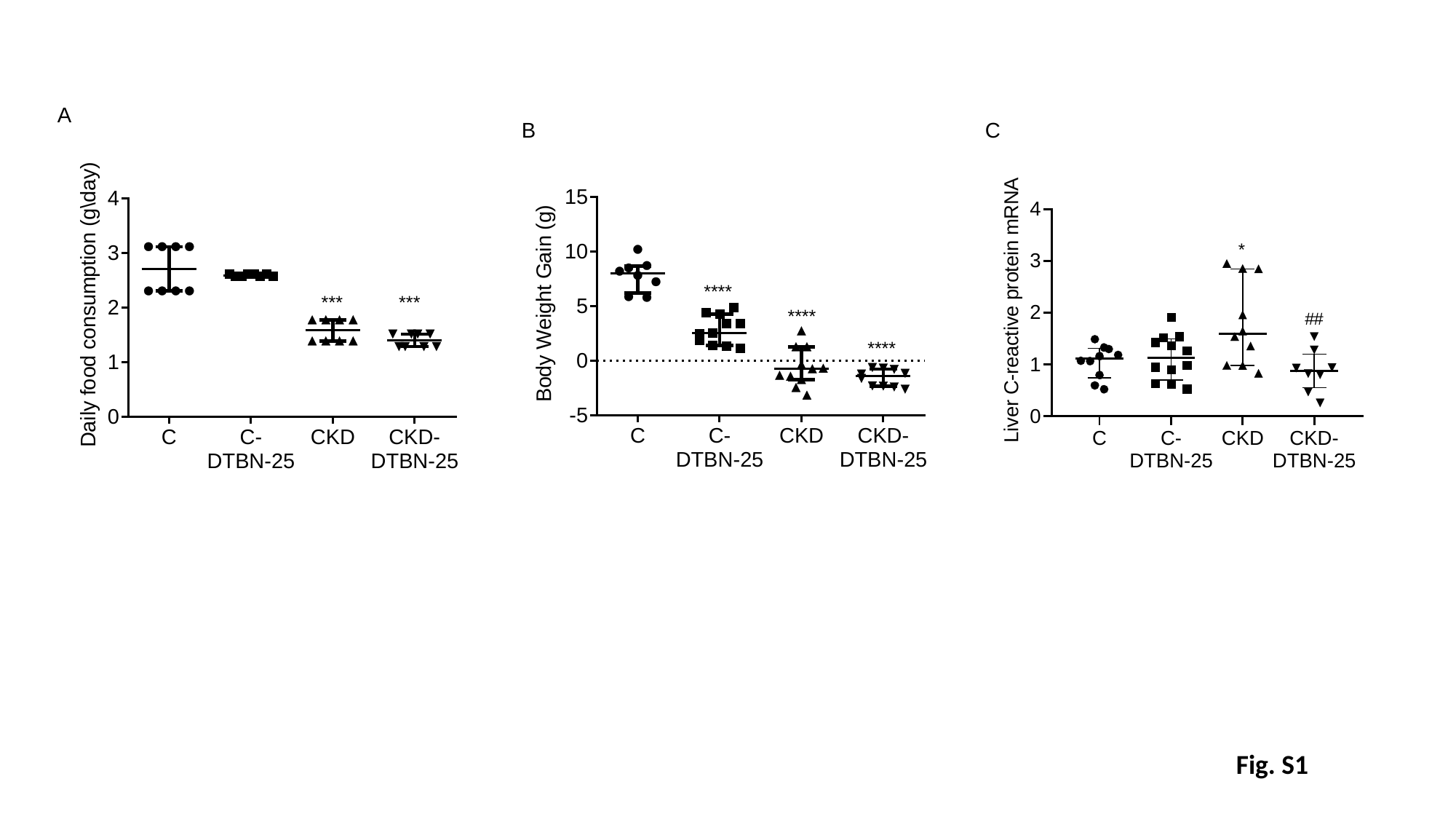

A
B
C
Fig. S1

Supplement: Supplementary file 1 — Supplementary Figure S1. [file 41598_2024_58055_MOESM1_ESM.pptx]

## Slide 1
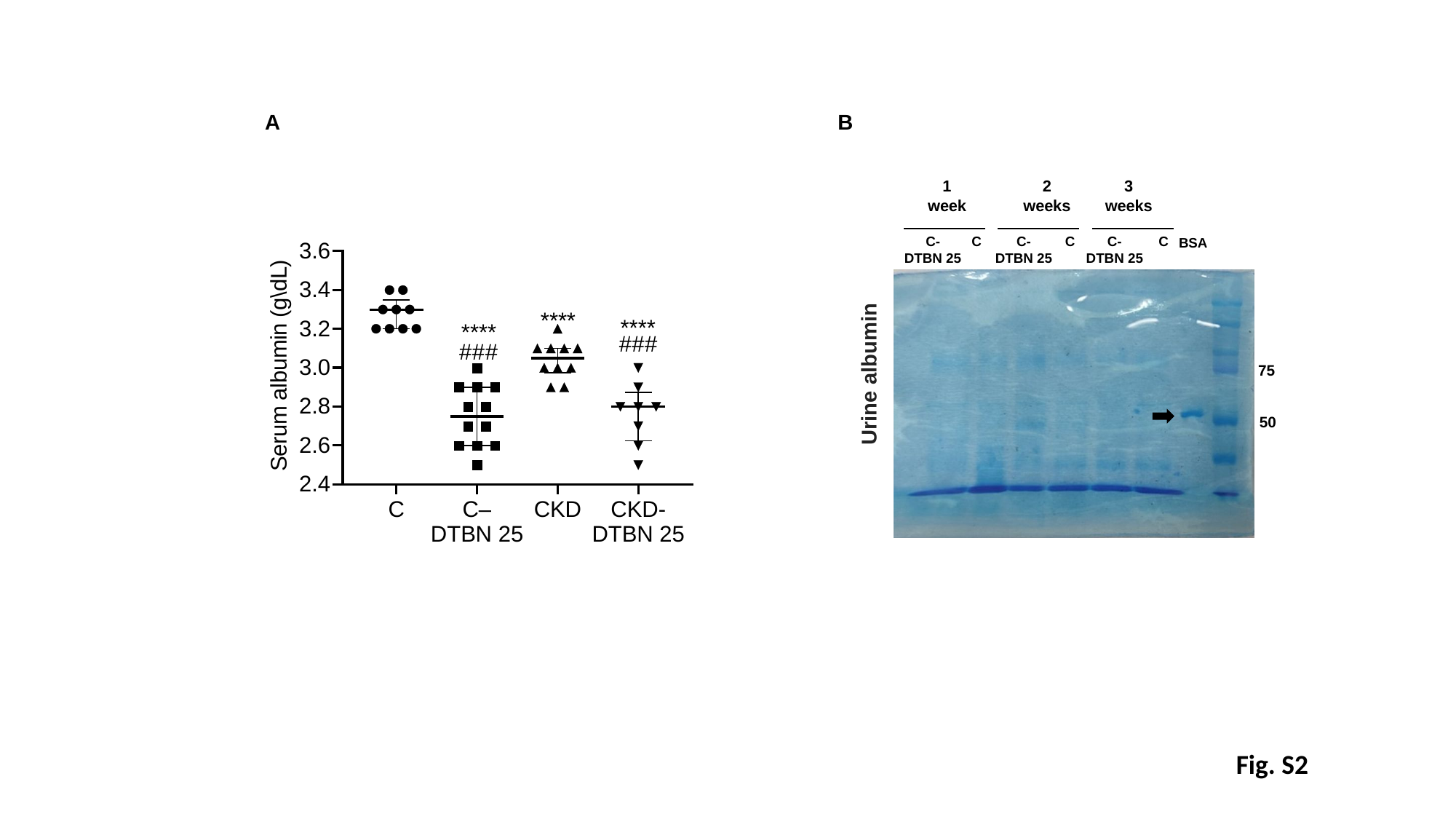

B
A
1
week
2
weeks
3
weeks
C-
DTBN 25
C
C-
DTBN 25
C
C-
DTBN 25
C
BSA
75
Urine albumin
50
Fig. S2

Supplement: Supplementary file 2 — Supplementary Figure S2. [file 41598_2024_58055_MOESM2_ESM.pptx]

## Slide 1
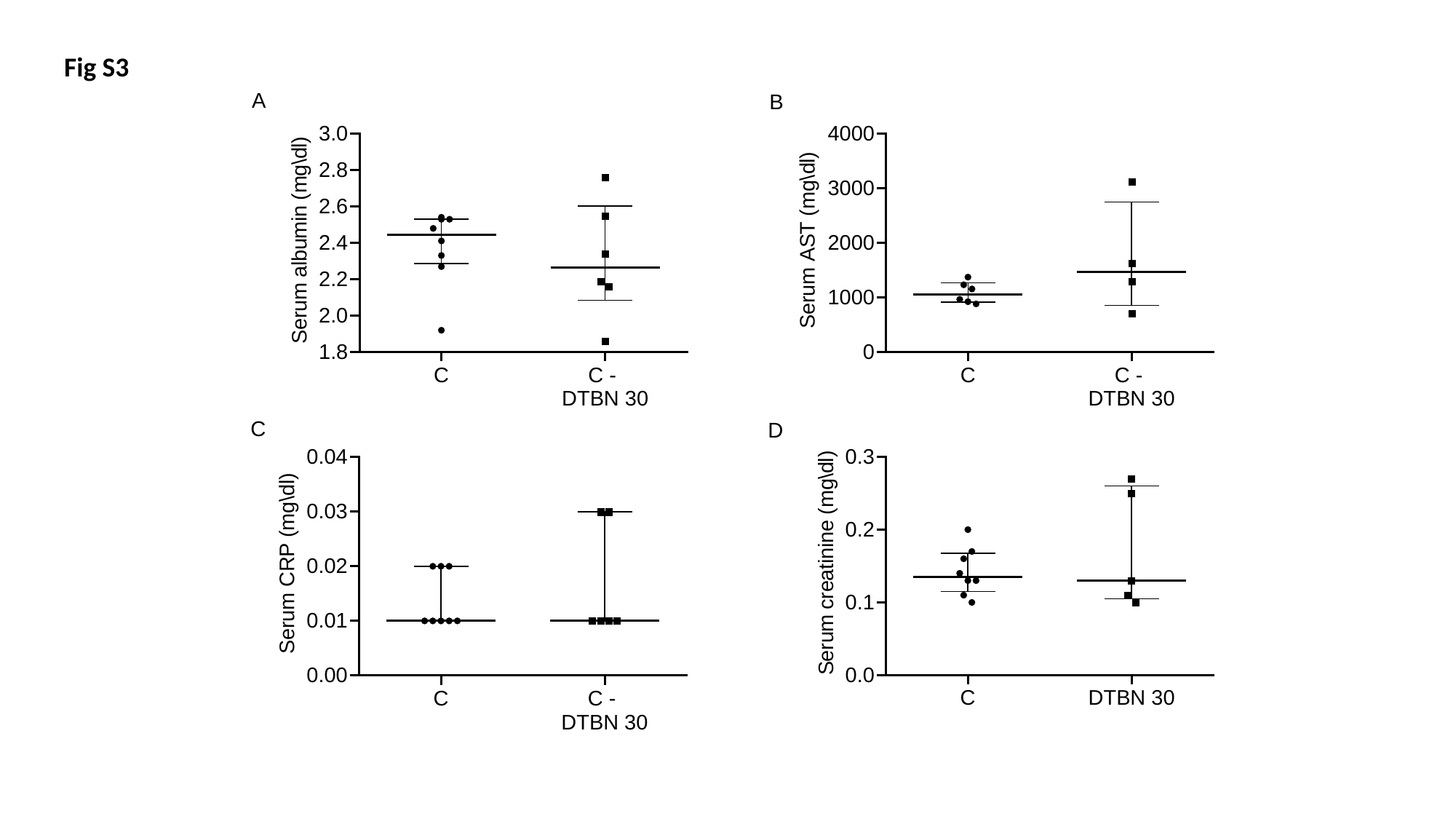

Fig S3
A
B
C
D

Supplement: Supplementary file 3 — Supplementary Figure S3. [file 41598_2024_58055_MOESM3_ESM.pptx]

## Slide 1
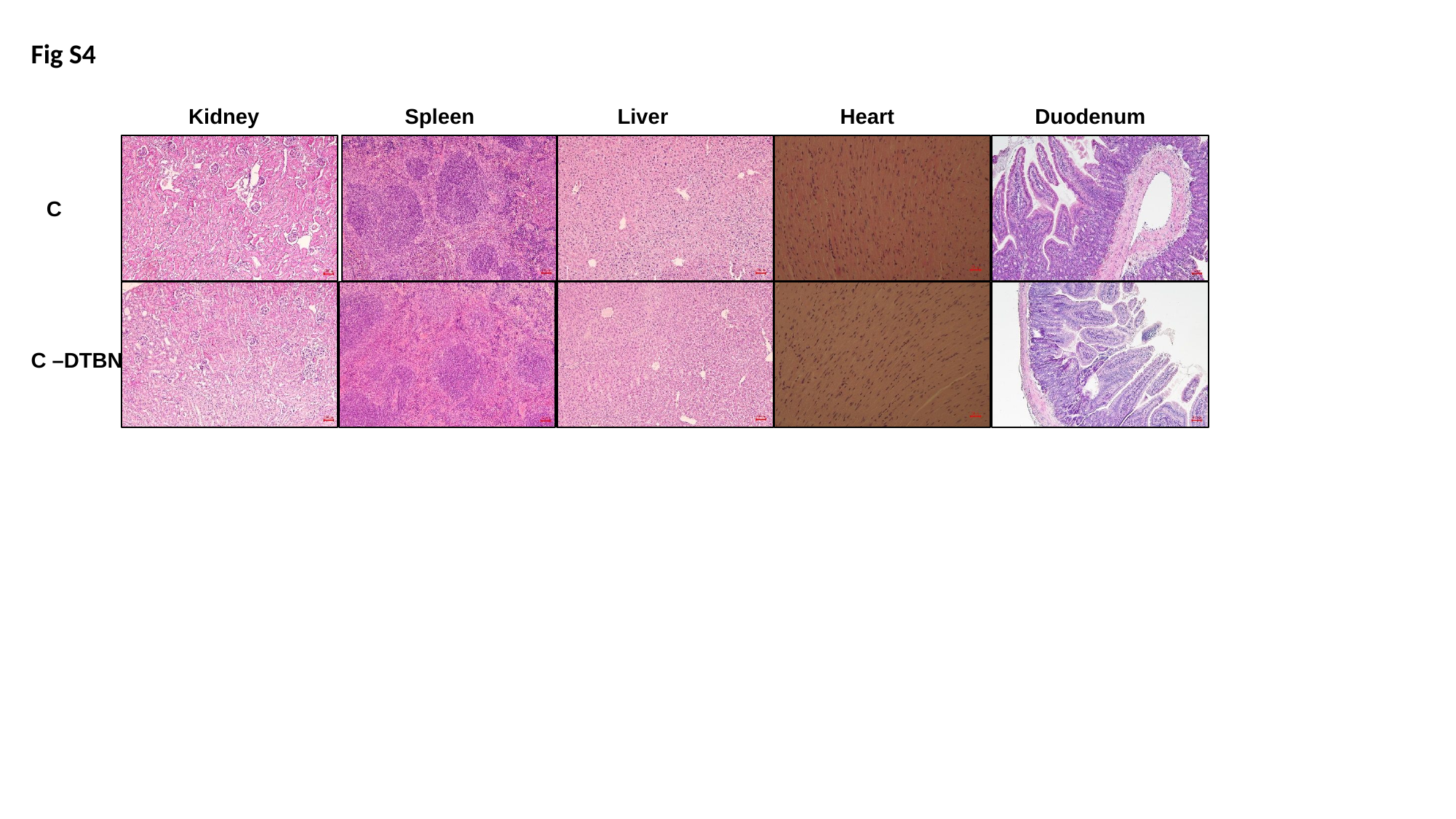

Fig S4
Liver
Heart
Duodenum
Kidney
Spleen
C
C –DTBN 30

Supplement: Supplementary file 4 — Supplementary Figure S4. [file 41598_2024_58055_MOESM4_ESM.pptx]

## Slide 1
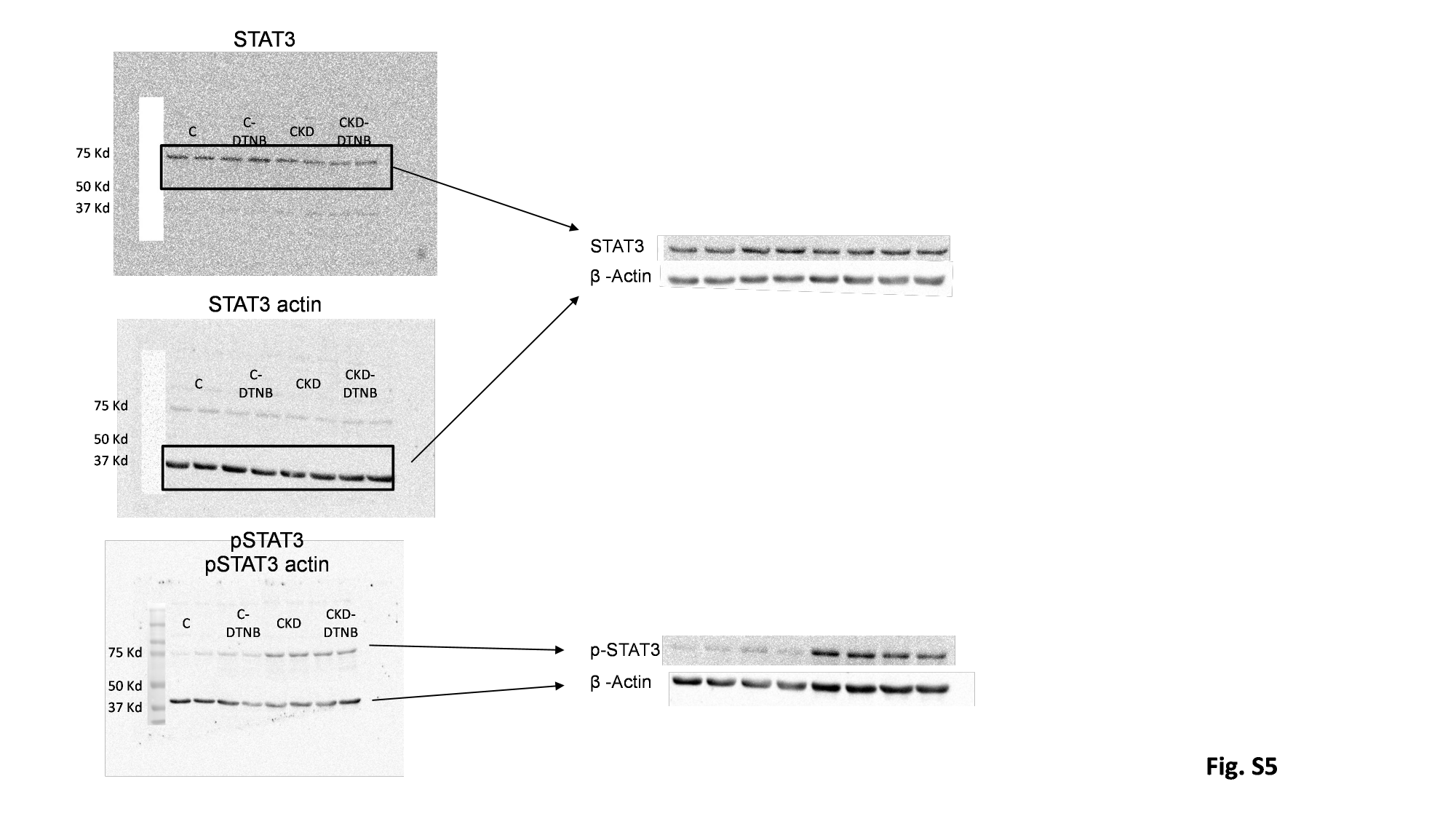

Supplement: Supplementary file 5 — Supplementary Figure S5. [file 41598_2024_58055_MOESM5_ESM.pptx]

## Slide 1
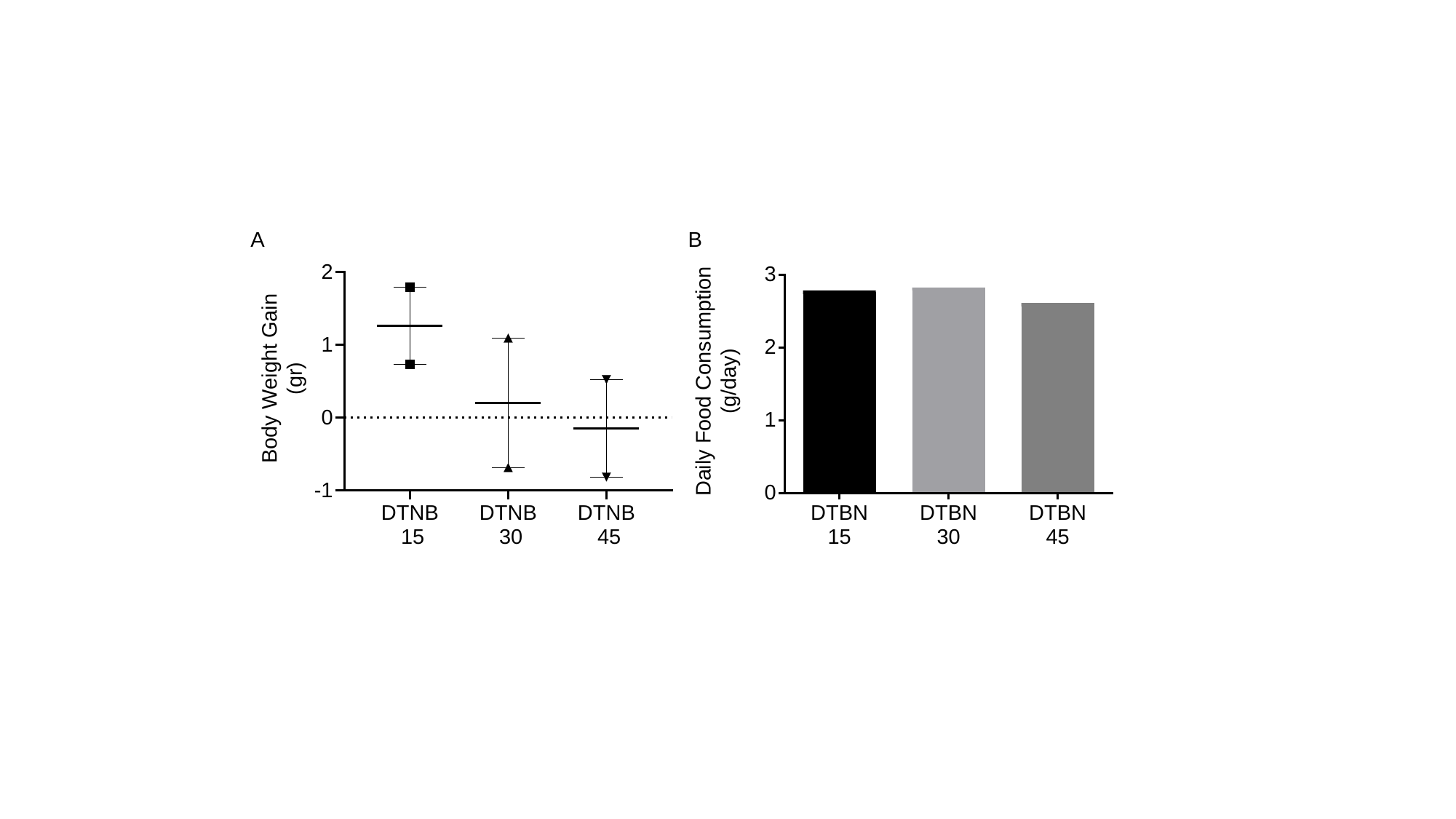

A
B

Supplement: Supplementary file 6 — Supplementary Figure S6. [file 41598_2024_58055_MOESM6_ESM.pptx]

## Slide 1
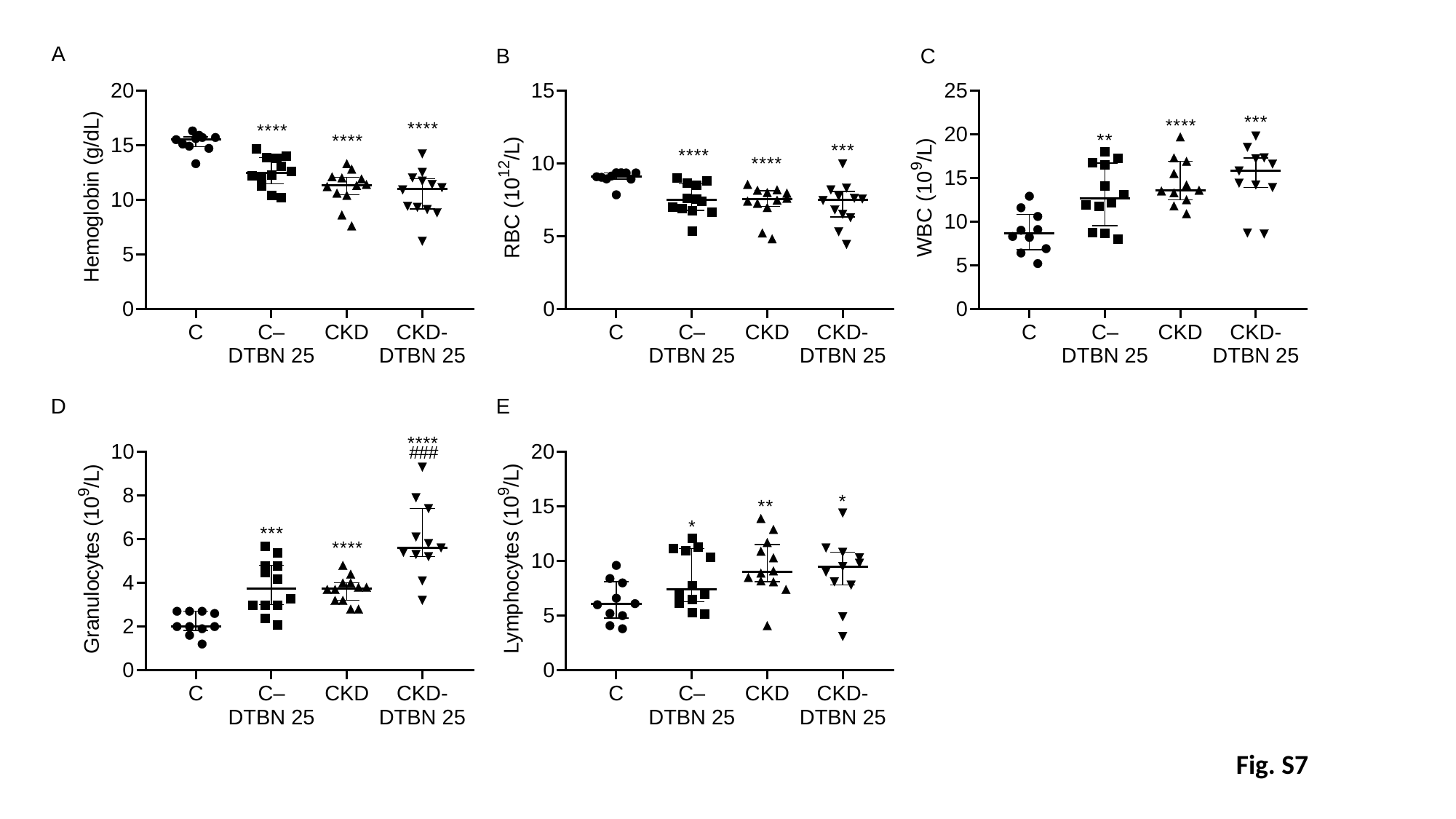

A
C
B
D
E
Fig. S7

Supplement: Supplementary file 7 — Supplementary Figure S7. [file 41598_2024_58055_MOESM7_ESM.pptx]
